# The role of vaccination in a model of asset pricing during a pandemic

**DOI:** 10.1371/journal.pone.0266511

**Published:** 2022-04-21

**Authors:** Yuta Saito

**Affiliations:** Graduate School of Economics and Business, Hokkaido University, Sapporo, Japan; University of Almeria, SPAIN

## Abstract

This paper examines the effect of pandemic vaccination on asset prices in a simple asset pricing model à la Lucas 1978. In this model, asset prices depend on susceptible individuals’ saving motives to insure against a reduction in labour income due to getting they get the virus. Hence distributing vaccine reduces precautionary saving motives and asset prices. This implies that reducing the income gap between susceptible and infected individuals, such as by cash handouts, eases the negative effect of vaccine supply on asset prices.

## 1 Introduction

This study examines the effect of vaccination during a pandemic on asset prices and its economic implications. We employ a simple susceptible-infected-recovered-deceased (SIRD) model [1] with an economic foundation à la Lucas [2]. In each period, individuals receive a state-contingent labour endowment. We assume that because infected individuals are sick, they receive less labour endowment than susceptible and recovered individuals. Distributing vaccines changes the status of a proportion of susceptible into recovered individuals in the next period.

The main results are two folds. First, an increase in vaccine supply decreases asset prices. Increasing vaccine distribution reduces the susceptible individuals’ saving motive to insure against future reduction in labour endowment. Hence, as more vaccines are distributed, the asset prices fall.

Second, cash handouts reduce the negative impact of vaccine supply on asset prices. The level of precautionary saving motives depends on the difference in the labour endowments of susceptible and infected individuals—a higher gap induces more saving motives. Thus, reducing the income gap, such as cash handouts to infected individuals, decreases the negative impact of vaccine supply on asset prices. We also show that equal amount of cash handouts to every individual reduces the negative effect of vaccine supply when the utility functions are concave.

Several studies have theoretically investigated asset prices during a pandemic. Studies such as [3–5] investigate the effects of the risk of rare disasters on asset markets. Caballero and Simesk [6] analyse the impact of central banks’ asset purchases on asset markets during a pandemic.

Saito and Sakamoto [7] study the effect of lockdown policies on asset prices. In the model, lockdown policies reduce precautionary savings by decreasing susceptible agents’ probability of getting infected in the future. They theoretically and empirically show that strengthening lockdown measures decreases asset prices at the time of implementation. Similarly, Toda [8] investigates the impact of the COVID-19 pandemic on an asset pricing model and discovers a negative link between stock prices and the number of infected agents; Detemple [9] investigates an asset pricing model and finds that during a pandemic, stock prices and interest rates react cyclically.

Several empirical studies have been conducted to investigate the financial markets during the COVID-19 pandemic. These studies include [10–17]. [13] contend that stock market volatility during the COVID-19 pandemic is primarily the result of government interventions, such as lockdowns, company closures, and direct cash transfers. This paper is also related with the literature on the macroeconomic impacts of pandemic policies, such as [18–31].

## 2 Model

This section illustrates our modelling of a pandemic and describes the individual economic behaviours in equilibrium.

### 2.1 Pandemic and vaccination

We study asset prices in a modified version of the SIRD epidemic model. Periods are indexed by *t*. In each period *t*, population (*N*) is divided into four groups—susceptible (*S*_*t*_) infected (*I*_*t*_), recovered individuals (*R*_*t*_), and deceased individuals (*D*_*t*_). Hence

$$\begin{array}{c} N=S_{t}+I_{t}+R_{t}+D_{t}, \end{array}$$ (1)

where we normalise *N* to be 1. Susceptible individuals have not had immunity to the virus; infected individuals have been infected and not recovered or dead yet; recovered individuals are either those who had recovered from the disease or have been vaccinated; deceased individuals have died due to the virus.

In each period *t*, susceptible individuals will be infected at a rate of *δ* > 0 and be vaccinated at a rate of *τ*_*t*_ ≥ 0; infected individuals will recover with probability *γ* > 0 and will die with probability *ρ* > 0. Here, we assume that the parameters are constant as it does not affect the implication of our main results. In reality, the emergence of a variant of the virus can change the parameters. The laws of motions are as follows:

$$\begin{array}{c} S_{t+1}=\left(1-\tau_{t}-\delta I_{t}\right)S_{t}, \end{array}$$ (2)

$$\begin{array}{c} I_{t+1}=\left(1+\delta S_{t}-\gamma -\rho \right)I_{t}, \end{array}$$ (3)

$$\begin{array}{c} R_{t+1}=R_{t}+\gamma I_{t}+\tau_{t}S_{t}. \end{array}$$ (4)

$$\begin{array}{c} D_{t+1}=D_{t}+\rho I_{t}. \end{array}$$ (5)

Note that the laws of motions 2–4 coincide with the standard SIR model if *τ*_*t*_ = 0 for all *t*.

### 2.2 Economy

The economy is based on the asset pricing model of Lucas [2]. The only assets in the economy are identical infinitely-lived trees. The trees generate dividends (fruits) that cannot be stored. In period *t*, there are *k*_*t*_ trees, with each generating *d*_*t*_ > 0 dividend. In period *t*, there are *k*_*t*_ trees, with each generating *d*_*t*_ > 0 dividend. Individuals in state *θ*_*t*_ ∈ {*S*, *I*, *R*, *D*} at *t* face the following budget constraint:

$$\begin{array}{c} c_{t}\left(\theta_{t}\right)+p_{t}k_{t+1}\left(\theta_{t}\right)=w\left(\theta_{t}\right)+\left(p_{t}+d_{t}\right)k_{t}\left(\theta_{t-1}\right), \end{array}$$ (6)

where *c*_*t*_ denotes consumption, *p*_*t*_ is the market price of a tree, and *w*(*θ*_*t*_) is nonstorable exogenous labour endowment. We assume that infected individuals receive a smaller amount of labour endowment than susceptible and recovered individuals; susceptible and recovered individuals receive the same amount of labour endowment, i.e. *w*_*t*_(*S*) = *w*_*t*_(*R*) > *w*_*t*_(*I*) ≥ *w*_*t*_(*D*) for all *t*; deceased individuals cannot consume, save or earn, i.e *c*_*t*_(*D*) = *w*_*t*_(*D*) = *k*_*t*+1_(*D*) = 0 for all *t*.

Let $q_{t+1}^{\theta_{t+1}|\theta_{t}}$ be the probability that an individual in state *θ*_*t*_ at period *t* will be in state *θ*_*t*+1_ at period *t* + 1. Then the laws of motions 2–4 yield $q_{t+1}^{S|S}=(1-\tau_{t}-\delta I_{t})$, $q_{t+1}^{I|S}=\delta I_{t}$, $q_{t+1}^{R|S}=\tau_{t}$, $q_{t+1}^{D|S}=0$, $q_{t+1}^{S|I}=0$, $q_{t+1}^{I|I}=1-\gamma -\rho$, $q_{t+1}^{R|I}=\gamma$, $q_{t+1}^{D|I}=\rho$, $q_{t+1}^{S|R}=0$, $q_{t+1}^{I|R}=0$, $q_{t+1}^{R|R}=1$, $q_{t+1}^{D|R}=0$, $q_{t+1}^{S|D}=0$, $q_{t+1}^{I|D}=0$, $q_{t+1}^{R|D}=0$, and $q_{t+1}^{D|D}=1$. With these probability measures, the expected intertemporal utility of individuals at *t* is as follows:

$$\begin{array}{c} E_{t}[\sum_{\omega =0}^{\infty }\beta^{\omega }[u(c_{t+\omega }\left(\theta_{t+\omega }\right))]], \end{array}$$ (7)

where *β* ∈ (0, 1) is the discount factor; $E_{t}$ is the expectation operator at *t*; *u* is an instant utility from consumption, which is increasing, concave, twice continuously differentiable, and *u*(0) = 0. Individuals maximise the intertemporal utility 7 subject to the budget constraint 6. The first-order conditions yield the following Euler equation:

$$\begin{array}{c} u^{\prime }\left(c_{t}\left(\theta_{t}\right)\right)=\beta E_{t}[u^{\prime }\left(c_{t+1}\left(\theta_{t+1}\right)\right)(\frac{p_{t+1}+d_{t+1}}{p_{t}})]. \end{array}$$ (8)

Since all endowments are nonstorable, in equilibrium, the aggregate dividend and income are all consumed—it holds that *S*_*t*_*c*_*t*_(*S*) + *I*_*t*_*c*_*t*_(*I*) + *R*_*t*_*c*_*t*_(*R*) = *d*_*t*_*k*_*t*_ + *w*_*t*_, where *k*_*t*_ = *S*_*t*_*k*_*t*_(*S*) + *I*_*t*_*k*_*t*_(*I*) + *R*_*t*_*k*_*t*_(*R*) and *w*_*t*_ = *S*_*t*_*w*(*S*) + *I*_*t*_*w*(*I*) + *R*_*t*_*w*(*R*). To obtain the results, we assume that the number of susceptible individuals is large enough at the initial period *t*, i.e. *S*_*t*_ ≈ *N*.

## 3 Results

Under the assumption of *S*_*t*_ ≈ *N*, arranging the Euler Eq 8 in equilibrium yields:

$$p_{t}\approx {\tilde{p}}_{t}=\frac{m}{u^{\prime }\left(c_{t}^{S}\right)},$$

where

$$\begin{array}{cc} m & =\beta (\sum_{\theta_{t+1}\in \{S,I,R\}}q_{t+1}^{\theta_{t+1}|S}u^{\prime }\left(c_{t+1}\left(\theta_{t+1}\right)\right))d_{t+1}\\ & +\beta^{2}(\sum_{\theta_{t+1}\in \{S,I,R\}}q_{t+1}^{\theta_{t+1}|S}\sum_{\theta_{t+2}\in \{S,I,R\}}q_{t+2}^{\theta_{t+2}|\theta_{t+1}}u^{\prime }\left(c_{t+2}\left(\theta_{t+2}\right)\right))d_{t+2}\\ & +\beta^{3}(\sum_{\theta_{t+1}\in \{S,I,R\}}q_{t+1}^{\theta_{t+1}|S}\sum_{\theta_{t+2}\in \{S,I,R\}}q_{t+2}^{\theta_{t+2}|\theta_{t+1}}\sum_{\theta_{t+3}\in \{S,I,R\}}q_{t+3}^{\theta_{t+3}|\theta_{t+2}}u^{\prime }\left(c_{t+3}\left(\theta_{t+3}\right)\right))d_{t+3}+\cdots . \end{array}$$

Note that since $q_{t}^{D|I}u\left(c_{t}\left(D\right)\right)=0$ for all *t*, *m* is independent from $q_{t}^{D|I}$ and *u*(*c*_*t*_(*D*)) for all *t*. Asset prices are determined by the expected value of the stream of future endowments. The vaccination measures (*τ*_*t*_, *τ*_*t*+1_, ⋯) affects asset prices by impacting the transition probabilities. Intuitively, asset prices reflect the degree of susceptible individuals’ precautionary saving motives. If individuals get infected, they receive less labour income; thus, they have the incentive to insure against the possibility of getting infected.

**Proposition 1**. *A higher vaccination measure decreases the current asset prices*:

$$\frac{d{\tilde{p}}_{t}}{d\tau_{t}}<0.$$

***Proof***. *Since* $\frac{dq_{t+1}^{S|S}}{d\tau_{t}}=-1$, $\frac{dq_{t+1}^{I|S}}{d\tau_{t}}=0$ *and* $\frac{dq_{t+1}^{R|S}}{d\tau_{t}}=1$, *we have*:

$$\begin{array}{cc} \frac{dm}{d\tau_{t}} & =\underset{\Delta_{t+1}=0}{\underset{︸}{\beta (u^{\prime }\left(c_{t+1}\left(R\right)\right)-u^{\prime }\left(c_{t+1}\left(S\right)\right))d_{t+1}}}+\underset{\Delta_{t+2}<0}{\underset{︸}{\beta^{2}(u^{\prime }\left(c_{t+2}\left(R\right)\right)-\sum_{\theta_{t+2}\in \{S,I,R\}}q_{t+2}^{\theta_{t+2}|\theta_{t+1}}u^{\prime }\left(c_{t+2}\left(\theta_{t+2}\right)\right))d_{t+2}}}\\ & +\underset{\Delta_{t+3}<0}{\underset{︸}{\beta^{3}(u^{\prime }\left(c_{t+3}\left(R\right)\right)-\sum_{\theta_{t+2}\in \{S,I,R\}}q_{t+2}^{\theta_{t+2}|\theta_{t+1}}\sum_{\theta_{t+3}\in \{S,I,R\}}q_{t+3}^{\theta_{t+3}|\theta_{t+2}}u^{\prime }\left(c_{t+3}\left(\theta_{t+3}\right)\right))d_{t+3}}}+\cdots \end{array}$$

*c*_*t*+1_(*S*) = *c*_*t*+1_(*R*) *implies that* Δ_*t*+1_ = 0. *Since c*_*t*_(*D*) < *c*_*t*_(*I*) < *c*_*t*_(*R*) *for all t and u is concave, we have u*′(*c*_*t*_(*D*)) > *u*′(*c*_*t*_(*I*)) > *u*′(*c*_*t*_(*R*)) *for all t. Since* $q_{t+1}^{I|S}$ *and* $q_{t+1}^{D|I}$ *are both positive for all t, we obtain* Δ_*t*+*ω*_ < 0 *for all ω* ≥ 2. *Since* $u^{\prime }\left(c_{t}^{S}\right)>0$, *we obtain the result*.

An increase in the vaccination measure (*τ*_*t*_) reduces susceptible individuals’ possibility of getting infected ($q_{t+1}^{I|S}$). The reduction in $q_{t+1}^{I|S}$ means susceptible individuals have a smaller risk of experiencing a reduction in income endowment. Therefore, susceptible individuals have less precautionary saving motives, which decrease the demand for assets and those prices.

**Corollary 1**. *As the income gap between susceptible and infected individuals (i.e*. *w*(*S*) − *w*(*I*)) *decreases, the impact of vaccination on asset prices (i.e*. $|\frac{d{\tilde{p}}_{t}}{d\tau_{t}}|$) *decreases. If w*(*S*) = *w*(*I*), *then* $\frac{d{\tilde{p}}_{t}}{d\tau_{t}}=0$.

Susceptible individuals’ precautionary saving motives depend on the income gap between susceptible and infected individuals; hence, a smaller income gap reduces their precautionary saving motives. Thus, the demand for the asset and its price decreases.

In an extreme scenario where the income gap is zero, *w*(*S*) = *w*(*I*), the vaccination measure does not affect asset prices. In such a case, the state of individuals does not affect their consumption levels; thus, the susceptible ones do not have precautionary saving motives. Hence, the vaccination measure does not affect individuals’ consumption and asset prices.

Corollary 1 implies that a simple cash handout policy can eliminate the negative effect of vaccination on asset prices. Let suppose individuals receive an unstorable cash handout, *b*_*t*_ > 0, which is irrelevant to individuals’ states. Now the budget constraint is given by:

$$c_{t}\left(\theta_{t}\right)+p_{t}k_{t+1}\left(\theta_{t}\right)=b_{t}+w\left(\theta_{t}\right)+\left(p_{t}+d\right)k_{t}\left(\theta_{t-1}\right).$$

**Corollary 2**. $\frac{d|(\frac{d{\tilde{p}}_{t}}{d\tau_{t}})|}{db_{t}}<0$

***Proof***. *Since individuals cannot store b*_*t*_, *we have c*_*t*_(*θ*_*t*_) = *b*_*t*_ + *d*_*t*_*k*_*t*_ + *w*(*θ*_*t*_). *u*″ < 0 *implies* $\frac{du^{\prime }\left(c_{t}\left(\theta_{t}\right)\right)}{db_{t}}<0$. *u*′ > 0, *u*″ < 0, *and w*(*R*) > *w*(*I*) *imply* $\left|\frac{du^{\prime }\left(c_{t}\left(R\right)\right)}{db_{t}}|=|\frac{du^{\prime }\left(c_{t}\left(S\right)\right)}{db_{t}}|<|\frac{du^{\prime }\left(c_{t}\left(I\right)\right)}{db_{t}}\right|$. *The desired result follows*.

Intuitively, lump-sum cash handouts reduce the marginal utility gap between susceptible and recovered individuals, which decreases precautionary saving motives. However, the result depends on the assumption that infected individuals can freely consume their endowments. If we assume that the health impact of virus is more severe and infected individuals cannot participate in economic activities (i.e. *c*_*t*_(*I*) = 0), we have $\frac{du^{\prime }\left(c_{t}\left(I\right)\right)}{db_{t}}=0$ and $\frac{du^{\prime }\left(c_{t}\left(S\right)\right)}{db_{t}}=\frac{du^{\prime }\left(c_{t}\left(R\right)\right)}{db_{t}}<0$, resulting in $\frac{d(|\frac{d{\tilde{p}}_{t}}{d\tau_{t}}|)}{db_{t}}>0$.

## 4 Conclusion

This study investigates the impacts of vaccination on asset prices in a simple model à la Lucas [2]. The main finding is that a higher vaccine supply decreases asset prices by reducing susceptible individuals’ saving motives to insure against the decline in labour income due to getting the virus. The result implies that reducing the income gap between susceptible and infected individuals mitigates the negative impact of vaccine supply’ on asset prices. We also show that distributing an equal amount of monetary transfers reduces the negative effect of vaccine supply.
